# A Comparative Study of Magnetic Properties of Large Diameter Co Nanowires and Nanotubes

**DOI:** 10.3390/nano8090692

**Published:** 2018-09-06

**Authors:** Jose Angel Fernandez-Roldan, Dieivase Chrischon, Lucio Strazzabosco Dorneles, Oksana Chubykalo-Fesenko, Manuel Vazquez, Cristina Bran

**Affiliations:** 1 Instituto de Ciencia de Materiales de Madrid, CSIC, 28049 Madrid, Spain; dieivase@gmail.com (D.C.); oksana@icmm.csic.es (O.C.-F.); mvazquez@icmm.csic.es (M.V.); 2 Physics department, Federal University of Santa Maria, RS 97105-900, Brazil; lsdorneles@gmail.com

**Keywords:** magnetic nanowires and arrays, magnetic nanotubes, magnetization reversal, coercivity and remanence angular dependence, micromagnetic simulations

## Abstract

A comparative study of the magnetic properties of the arrays of Co nanowires and nanotubes with large external diameters (180 nm) has been carried out. The nanowires/nanotubes were grown by electrodeposition into the self-assembled pores of anodic alumina membranes. The experimental study of their magnetic behavior was focused on the angular dependence of hysteresis loops and their parameters. In both nanowire and nanotube arrays, from the analysis of experimental data, effective longitudinal magnetic anisotropy is concluded, which is stronger in the case of the nanotube array. In addition, the extremely small remanence observed for all loops indicates the important role played by magnetostatic interactions. Micromagnetic simulations were first performed considering intrinsic shape and magnetocrystalline anisotropy terms, together with an effective easy-plane anisotropy to account for those magnetostatic interactions. A qualitative agreement between experiments and simulations is found despite the complexity introduced by the intrinsic and extrinsic array properties (i.e., large diameters, grain structure, and array configuration). In addition, simulations were also carried out for individual nanowire/nanotube with a particular emphasis to understand their differences at the remanence, due to pure geometry contribution.

## 1. Introduction

Cylindrical magnetic nanowires (NWs) and nanotubes are technologically promising and important nanostructures that are relevant for several applications: 3D magnetic recording, optoelectronics, sensors, actuators, spintronics (i.e., spin valves, magnetic tunnel junctions), logical devices, permanent magnets, barcode nanostructures catalysis, and bio and environmentally friendly applications (such as water purification or hyperthermia) [1,2,3,4,5,6,7,8,9,10,11,12,13]. The revolution symmetry of nanowires and nanotubes makes these systems suitable for domain-wall-based technologies. The magnetization dynamics in these nanostructures has interesting properties in the breaking of the inversion symmetry [14]. Several of these applications require a high net magnetic moment and an accurate control over the demagnetization process and stability. For that purpose, cobalt hcp stands out among other suitable materials due to its relatively large saturation magnetization and consequent high shape anisotropy, along with high magnetocrystalline anisotropy. As a consequence, a high energy product in Co nanowires has been obtained [15].

Regarding the synthesis of nanowires, the versatility of the fabrication process by electrodeposition allows the production of different Co nanowires and nanotubes with tailored properties. Co-based nanowires and nanotubes typically contain both fcc and bcc phases that coexist with the hcp phase and large monocrystalline grains with different growing directions [16]. Hcp Co-based nanowires can also be prepared in the monocrystalline phase where the easy axis of uniaxial magnetocrystalline anisotropy was found to be nearly perpendicular to the nanowire being controlled by deposition and temperature parameters [17,18].

With the aim to control the magnetization processes, nanowires with modulations in diameter or material composition are currently studied. One of most recent results on Co-based nanowires is the unidirectional propagation (i.e., magnetization ratchet) of the magnetization reversal irrespective of the longitudinal applied field direction [19]. Additionally, attractive for applications is the exchange bias obtained by the oxidation or coating of the inner surface in Co core-shell nanotubes, which offer additional tailoring capabilities due to their hollow geometry [13,20].

Regarding the theoretical description on individual nanowires, the high uniaxial magnetocrystalline anisotropy of the hcp phase in competition with the shape anisotropy leads to a strong dependence of the coercive field and remanence on the easy axis orientation [21] and on the particular granular structure in Co based nanowires [22]. Individual nanowires with cubic structures exhibit, typically, squared hysteresis loops, while the hcp nanowires with a perpendicular *c*-axis are characterized by a small remanence. Arrays of these nanostructures with large diameters (short interwire distances) are still challenging for calculation of the dipolar interwire interaction [17,23]. Some analytical models of the interaction between neighboring nanostructures typically assume constant magnetization and work for long range interactions only but they miss higher order contributions (than the dipole in the multipolar decomposition) from farther nanostructures or do not consider close inter-nanostructures distances [1,2].

The demagnetization process in both Co nanowires and nanotubes, dominated by the shape anisotropy or with easy axis almost parallel to the axis, typically starts by the nucleation of a domain wall at the nanowires ends [24]. At the coercive field, this domain wall is unpinned and propagates towards the opposite end of the nanowire. The reversal mode, and thus the nature of the domain wall, is determined by the material, but also by the diameter in the long nanowires (with an aspect ratio that is the length-diameter equal or larger than 10), and even with the wall thickness in nanotubes [1,25,26]. In the mentioned conditions, a transverse domain wall is generally obtained for a small diameter, while vortex domain walls are energetically favored for large diameters in long nanostructures [27]. Theoretically, the transition from a transverse to vortex domain wall for individual cubic Co-based nanowires takes place for 30 nm diameters [28], while experimentally it has been inferred to be between 20 and 40 nm from the angular measurements of the coercive field and analytical models in the arrays of nanowires [17] and the above 50 nm in arrays of Co nanotubes [24]. Recently, the reversal mode in tubular structures has been reported to be modified by dipolar interactions [29].

Co hcp nanowires with a perpendicular easy-axis have a completely different behavior and are characterized by a low remanence and magnetically soft properties due to the compensation between the magnetocrystalline and the shape anisotropies. Namely, the array of vortices with alternative chiralities has been modelled and imaged by the magnetic force microscopy in individual Co hcp and Co based nanowires [30]. Electron holography measurements also confirmed that these nanowires showed alternating vortex states at the remanence, leading to the proposal of the vortex-based media [31,32].

The direct observation of the inner and surface magnetization structures in geometrically modulated individual Co hcp nanowires, performed by the X-ray Magnetic Circular Dichroism with PhotoEmission Electron Microscopy (XMCD-PEEM) technique, also revealed a complex internal magnetization structure characterized by a collection of vortices with periodically alternating chiralities placed along the nanowire length at remanence [28]. In more recent works performed in Co-based nanowires, the presence of such vortex domains was confirmed together with transverse domains placed along the nanowire [33]. These domains are characterized by exhibiting the periodical alternation of the sense of the magnetization between contiguous domains, leading to a striped pattern [33]. These states were additionally obtained in modelling by energy relaxation from different initial configurations which corroborates their stability [33].

More recent theoretical studies in CoFe nanowires with large diameters (120 nm) even suggest the formation of non-trivial topological structures called skyrmions-tubes in nanowires during magnetization reversal [34]. Besides, a novel pinning mechanism has been described in nanowires with modulated diameters [22]. The nanotubes are envisaged to have basically similar magnetic properties [35] with the difference that the vortex domains should not have the axial magnetization corresponding to the vortex core.

In this work, we have synthesized Co nanowires and nanotubes with large diameters, 180 nm, and lengths around 6 μm. The nanowires and nanotubes structure has been determined, while embedded in the Anodic Aluminum Oxide(AAO) templates using Transmission Electron Microscopy (TEM) through Focus-Ion-Beam (FIB) sample preparation. The hysteresis loops of arrays have been measured by paying attention to the angular dependence, their coercivity, and remanence. There, magnetostatic interactions are considered to be very relevant. Consequently, micromagnetic simulations have been performed in nanowires and nanotubes where magnetostatic interactions are taken into account in the mean-field approach. To disentangle the influence of dipolar interactions from the pure geometrical effects on magnetic configurations, we also performed simulations for non-interacting objects. Our results show a larger “effective shape anisotropy” in nanotubes, as compared to nanowires, due to the influence of the inner surface.

## 2. Synthesis and Structural Characterization of Nanowires and Nanotubes

Arrays of Co nanowires and nanotubes were grown by electrodeposition inside the hexagonally self-assembled nanopores of alumina membranes. These membranes were prepared from within high-purity alumina foils (99.999% pure content [28,36] by hard anodization at 140 V in an oxalic acid aqueous solution containing 5 vol.% of ethanol at a temperature between 0 and 1 °C). After a hard anodization process, the aluminum traces were removed by wet chemical etching in a CuCl_2_=HCl aqueous solution and the pores were opened by immersion in 5% H_3_PO_4_ solution at 35 °C for 2 h, resulting in a membrane with pores open by both sides. The final pores have a diameter of about 180 nm and an interpore distance (center to center) of 320 nm.

A gold (Au) layer was sputtered on one side of the membrane to work as an electrode for the electrodeposition of Co. For long enough sputtering time, the Au nanolayer close the pores at the bottom allowing the growth of nanowires, while for short sputtering time, pores remain only partly closed determining the lateral growth of Co resulting in Co nanotubes. The nanostructures were prepared by electrodeposition using the electrolyte: CoSO_4_ · 7H_2_O (0.36 M) + H_3_BO_3_ (0.16 M). The pH value was kept constant at about 3.0. The length of the nanowires/nanotubes was determined by the total charge/time of the electrodeposition.

The morphology of nanowires/nanotubes in the membranes has been characterized by scanning and transmission electron microscopies imaging techniques. Their structure was determined with a commercial PANanalytical X’pert ProX-ray diffractometer (XRD) in a Bragg-Brentano geometry using the Cu Kα lines.

**Figure 1:**
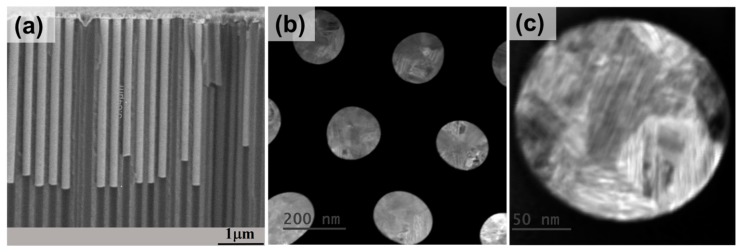
(**a**) Cross-section SEM image of AAO membrane filled with Co nanowires; (**b**) Top STEM image of the Co nanowires within the membrane; (**c**) closer look of a single nanowire presenting crystallites with different orientations.

The images presented in Figure 1 show the morphology and structure of nanowires while embedded into the AAO template. Cross-section Focus-Ion-Beam (FIB) sample preparation is convenient in the present case since it preserves the structure and the arrangement of the wires inside the AAO template [37]. The images show an almost full filling of the pores with nanowires, and they confirm, overall, the presence of hexagonally arranged nanowires with a large diameter of 180 nm, interwire distance of 320 nm, and a total length of approximately 6 µm. Figure 1a shows a lateral scanning electron microscopy (SEM) image for the array of nanowires, while Figure 1b depicts the STEM image of the top of the membrane filled with nanowires. The lengths of the nanowires are quite uniform, the shorter ones presented in the figure are NWs broken during the preparation of the sample for the SEM measurements. On the other hand, magnetic properties of individual nanowires become independent of the nanowire length aspect ratios length/diameter larger than 10 [21], as the shape anisotropy dominates the reversal process and is nearly independent for high aspect ratios. Furthermore, STEM bright and dark field images confirm the presence of multiple large crystal domains with multiple growing orientations of the crystal lattice (see Figure 1b,c). The observed crystal planes do not remain flat at large scales, which is interpreted as the presence of small multiple defects distributed in the crystal lattice, contributing to the main grain orientation. On the other hand, the high resolution electron micrographs obtained by transmission electron microscopy (TEM) (see Figure 1c) confirm again the presence of multiple crystal domains of different sizes with the presence of multiple twinned crystalline materials. The X-Ray diffraction measurements of Co nanowire arrays indicate the presence of hexagonal symmetry hcp crystals.

**Figure 2:**
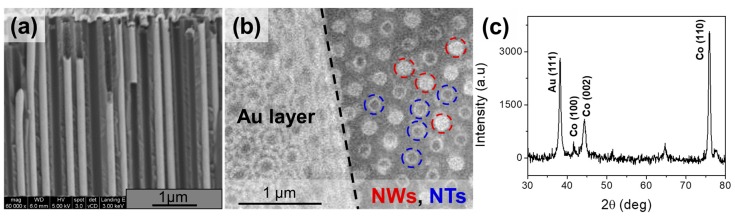
(**a**) Cross-section SEM image of AAO membrane filled with Co nanotubes; (**b**) top-view SEM image of nanotubes sample before and after a part of the Au layer was removed to image the nanostructures embedded into membrane; and (**c**) XRD pattern of the nanotubes within the AAO template whose peaks correspond to hcp Co phase and to the back sputtered Au layer.

In the case of Co nanotube array, in some cases, we observed that pores are not homogeneously filled. As an example, Figure 2a shows the SEM lateral view of the nanotubes inside the membrane. In Figure 2b we observed the presence of nanotubes together with some nanowires and vacancies (top view SEM images at the bottom side of the membrane after the Au layer was partially removed). The thickness of nanotubes has been estimated to be 20 to 40 nm. The XRD spectrum of the Co nanotubes embedded into the AAO template are shown in Figure 2c. The X-ray data shows, for both samples, nanowires and nanotube walls, the hcp phase of Co where the (110) peak strongly dominates the crystallographic structure. The hcp phase with texture in the (110) direction presents a strong magnetocrystalline anisotropy of hexagonal symmetry with the magnetization easy axis nearly perpendicular to the nanowire axis [18]. The XRD pattern also shows a strong peak close to 2θ= 38° which is assigned to fcc Au (111) coming from the back side of the sample. The other unmarked peaks belong to the Co oxides formed after the partial removal of Au layer.

## 3. Magnetic Characterization

The magnetic behavior of the nanowire/nanotube arrays were measured in a vibrating sample magnetometer (VSM) at room temperature under a maximum field of 1.8 T applied. The angular dependence of the magnetic response to the applied field from parallel to perpendicular orientation, with respect to the nanowires axis, was particularly preformed.

**Figure 3:**
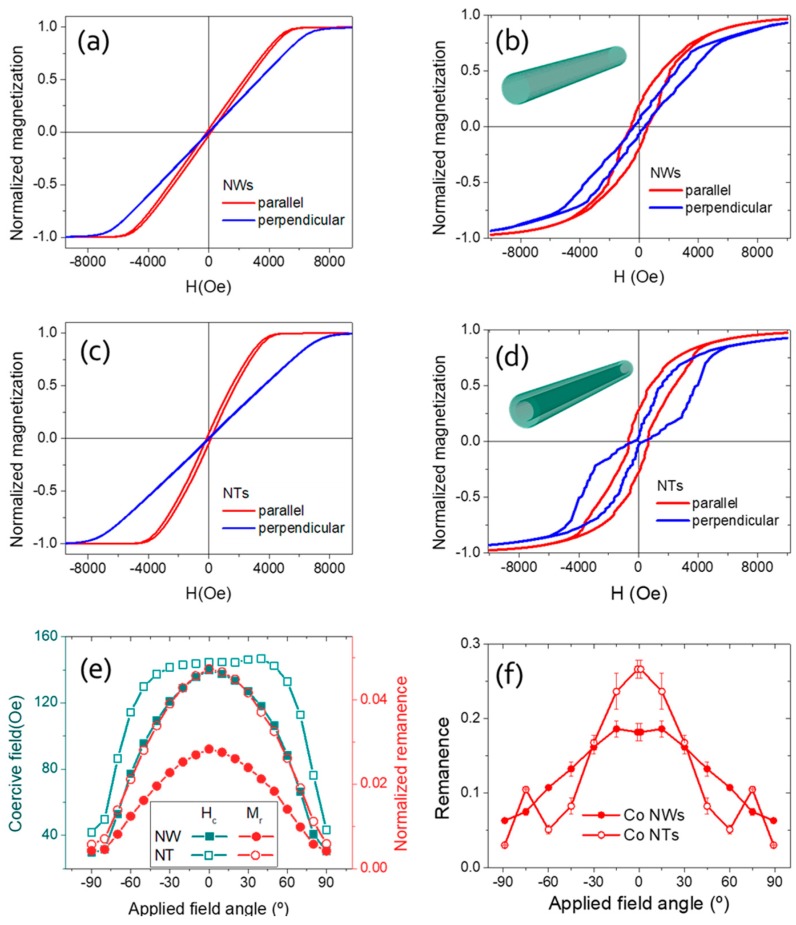
Experimental hysteresis loops for arrays of (**a**) nanowires and (**c**) nanotubes, respectively. Micromagnetic modelled loops of the arrays modelled in individual nanowire (**b**) and nanotube (**d**) with an effective ‘interaction’ anisotropy; (**e**) angular dependence of experimental coercive field and normalized remanence for nanowire and nanotube arrays; and (**f**) angular dependence of simulated remanence for nanowire and nanotube.

The hysteresis loops of arrays of nanowires and nanotubes were firstly measured under applied field parallel and perpendicular to the nanowire axis (See Figure 3a,c, respectively). In all the loops, we observe an almost constant susceptibility until nearly reaching magnetic saturation. The parallel hysteresis loops reach magnetic saturation at applied fields of H_s,para_ = 5.5 and 4.0 kOe for the nanowire and the nanotube arrays, respectively. For the perpendicular applied field, saturation fields are H_s,perp_ = 7.0 and 7.5 kOe for nanowire and nanotube arrays, respectively. All these data indicate, firstly, that in both nanowires and nanotubes there is an effective anisotropy favoring an easier magnetization along the axis of nanowires and nanotubes (i.e., larger susceptibility for parallel applied field). However, the role played by magnetostatic interactions seems to be really determinant as parallel loops show very small remanence. In addition, a measure of the effective axial anisotropy can be estimated from the differential saturation field (ΔH_s_ = H_s,para_ − H_s,perp_), which takes values of ΔH_s_ = 1.5 and 3.0 kOe for nanowire and nanotube arrays, respectively. Particular values of coercivity for nanowire and nanotube arrays are H_c_ = 138 and 35 Oe for parallel and perpendicular loops, respectively, while for nanotubes those values are, respectively, H_c_ = 143 and 43 Oe, denoting the comparatively larger axial anisotropy in nanotubes. In all the hysteresis loops the remanence takes very small values.

Further study has been performed as a function of the angle between the applied field and the nanowires/nanotubes axis from parallel to perpendicular orientations. Hysteresis loops for all intermediate directions show that nanowire and nanotube arrays are harder to magnetize in those directions than in parallel-applied fields. Figure 3e shows the experimental angular dependence of coercivity and remanence for nanowire and nanotube arrays. In the whole angular range, both coercivity and remanence show larger values for nanotubes than for nanowires. Particularly, the quite small values of the normalized remanence denote the significant role played by magnetostatic interactions, favoring an effective “in-plane” anisotropy (i.e., in the plane of the membrane). At first sight, the larger remanence of the nanotube sample can be attributed to a smaller influence of this magnetostatic effect due to a smaller magnetic volume of the nanotubes, as compared to the nanowires.

## 4. Micromagnetic Simulations and Discussion

For a better understanding of the different magnetic behavior of nanowires and nanotubes, and particularly of the role played by magnetostatic interactions and the shape effects, micromagnetic simulations of the hysteresis loops of Co nanowire/nanotube arrays have been calculated using the Object Oriented MicroMagnetic Framework (OOMMF) package [38] for different applied field directions with the nanowire/nanotube axis, θ, varied between 0° and 90°. The simulated samples have a length of 2 µm and a diameter of 180 nm. The material parameters used for these calculations are: Saturation magnetization μ_o_M_s_ = 1.75 T; exchange stiffness A_ex_ = 3.0 × 10^−11^ J/m; and uniaxial magnetocrystalline anisotropy constant K_u_ = 4.5 × 10^5^ J m^−3^ [27,39]. A polycrystalline structure was modelled by slicing the system in 70 layers of equal height along the nanowire length and setting the easy axis of the magnetocrystalline anisotropy at 75° with the nanowire/nanotube axis [30], and a random azimuthal angle in each layer (i.e., as a different “grain”). In addition to the magnetocrystalline anisotropy, an effective “interaction” anisotropy of the easy-plane type has been introduced to account for the magnetostatic (interwire) interaction of the ensemble of nanowires/nanotubes in the membrane. Such anisotropy with easy plane perpendicular (i.e., “in-plane”) to the nanowire axis was added with the anisotropy constant K_d_ = − F μ_o_M_s_^2^. Ideally, the parameter F should correspond to the filling factor of the array. However, the use of the value estimated for the ideal hexagonal array did not produce an agreement with experimental data which we attribute to various contributions such as the presence of nanowires in the nanotube array, partially filled pores, and the variation of the geometrical parameters such as nanowire diameters and interparticle distances. Consequently, F was used as a fitting parameter. Its estimation has been carried out by a subtle variation and tuning of the magnetic properties for both nanowire/nanotube arrays to get the closest loop shape to the experimentally measured, obtaining the value F = 0.07. Since this value is the same for nanowires and nanotubes, the real difference is coming from different magnetic volume, making the interaction strength smaller in nanotubes than in nanowires.

The simulated hysteresis loops for parallel and perpendicular applied field directions are presented in Figure 3b,d. The overall loop shapes match the experimental results reasonably. In both nanowire and nanotube, the parallel loop shows a larger susceptibility, which confirms the effective axial easy axis. Besides, the coercive field obtained in the parallel loop is larger than that of the perpendicular loop. However, the values obtained are overestimated. This is interpreted as a consequence of an overestimation of the dipolar interaction of a single nanowire with the surrounding ones (i.e., the additional anisotropy). The real magnetostatic coupling between the neighboring nanowires [40,41] is of the antiparallel nature and can promote a sequential reversal of neighboring nanowires, reducing the effective interaction anisotropy, which is neglected in our simulations. Moreover, the presence of the additional structural defects that would promote domain wall nucleation and decrease the coercive field is not considered in the model. Finally, the thermal activation effects that also decrease the coercivity (and to a smaller extend the remanence) are not taken into account.

The angular dependence of the remanence obtained by simulations is presented in Figure 3f. The monotonic decreasing behavior of the remanence of the array is qualitatively reproduced by the simulations with higher values in Figure 3f. Additional peaks in the remanence in the nanowires as a function of the applied field angle (not present in the experiment) correspond to the easy axis direction. The difference between experiment and simulations for the magnetic nanotubes may arise from the presence of some nanowires in the array, screening the predicted peaks. Overall, the simulations and experiments reasonably agree, although a discrepancy is found in absolute values due to the limitations of the model that otherwise also predicts larger remanence in nanotubes, as compared to nanowires.

**Figure 4:**
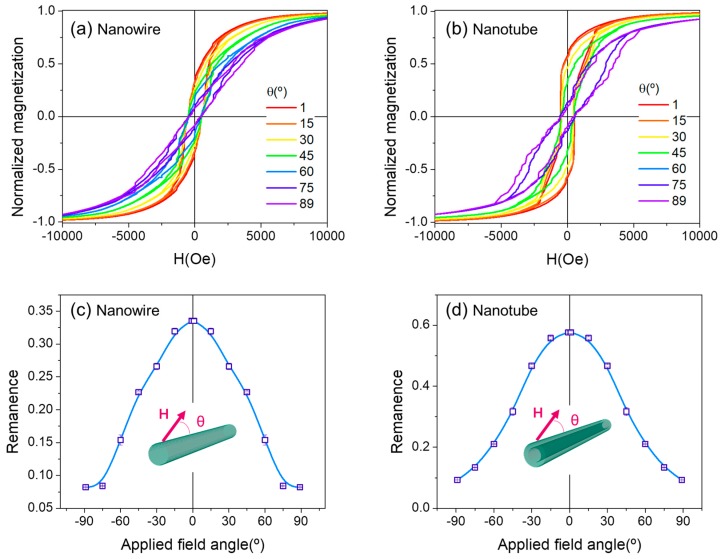
Simulated angular dependence of hysteresis loops for a single nanowire (**a**) and a single nanotube (**b**) and their respective remanence (**c**,**d**).

Finally, for a better understanding of the geometry factors governing the observed differences, apart from the magnetostatic interactions, we performed micromagnetic simulations on the angular dependence of hysteresis loops for individual nanowires and nanotubes (i.e., released from the membrane), i.e., with no interwire interactions (F = 0) (See Figure 4). Note that in the array the influence of the geometry (nanotube versus nanowire) is screened by the fact that the tubes have smaller magnetic volume and thus smaller magnetic interaction.

The hysteresis loops for the individual nanowire (Figure 4a) are rounded and evolves monotonically by increasing the applied field angle. However, for the nanotube, a two-fold reversal mechanism is evidenced particularly for the larger angles (Figure 4b). The remanence monotonically decreases with higher applied field angles in both nanowires/nanotubes (Figure 4c,d), but have different tendencies: Nanotubes present an inflection point close to 45º. Overall, nanotubes have larger remanence than nanowires.

**Figure 5:**
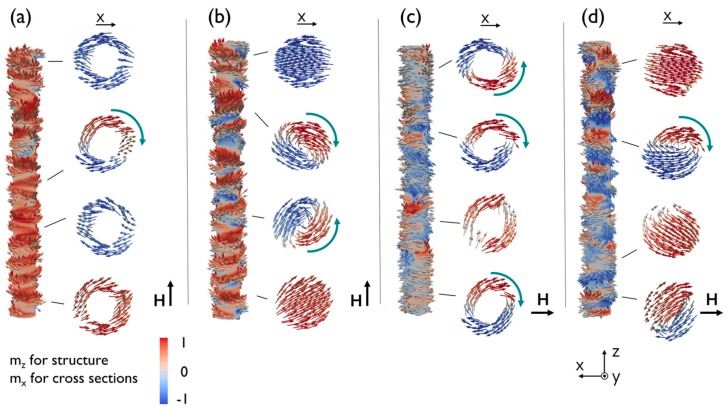
(Color online) From left to right, four figure compositions showing the magnetization configurations at remanence of single nanotubes (**a**,**c**) and nanowires (**b**,**d**) for parallel (**a**,**b**) and perpendicular (**c**,**d**) applied field orientations (as indicated by black arrows). On the left side of each composition, the nanowire/nanotube (the structure) is colored by the longitudinal m_z_ component of magnetization. On the right side of each composition, cross sections at the marked places are colored by the transverse component m_x_ of magnetization. Green arrows over vortex cross-sections indicate the vortex circulation.

In order to compare nanowire and nanotube systems and understand the remarked features, the simulated magnetization configurations at remanence are presented in Figure 5 for parallel and perpendicular applied field configurations. Red and blue colors correspond to the magnetization along the axial direction in the nanowire/nanotube images, but indicate the transverse magnetization component in the cross sections. As observed clearly, in the parallel configuration for the nanotube, the red color becomes more evident than for the nanowire, indicating more axial magnetization orientation. This is in agreement with the larger remanence of the nanotube, as compared to nanowires. Consequently, the larger remanence of nanotubes found experimentally is not due to magnetostatic interactions only, but has an important geometrical contribution.

Both the nanowire and nanotube exhibit sequences of vortex and transverse domains for parallel and perpendicular hysteresis cycles. Note that the domains present a spiral structure similar to the mixture of perpendicular and vortex domains reported recently in CoNi alloys with smaller diameters and confirmed by direct experimental measurements of magnetization textures by XMCD-PEEM techniques [33].

Additionally, for parallel applied fields, the vortices cores in the nanowire shows an asymmetrical deformation already observed previously and attributed to the transverse magnetocrystalline anisotropy [28]. The nanowires are characterized by a larger number of transverse domains, while the nanotubes have a larger number of vortices. The vortices in the nanotube, lacking any core, reveal that the pole avoidance on the inner surface promotes the flux closure curling mostly tangent on the internal surface. Overall, a larger magnetization component along the longitudinal direction is found in nanotubes than in nanowires for parallel-applied fields, consistent with the larger magnetization previously found. In the cross-sections, the “onion” states [42], typical for the planar rings with non-net magnetization, are visible.

For perpendicular applied fields, the reverse situation is found: Not only the magnetization texture in the nanostructure consists mostly of sequences of vortices along its length (See Figure 5c,d), but also, the extension of these vortices is slightly larger than that observed for the parallel field in each nanostructure and their dimensions are longer in the nanotube than in the nanowire. As a confirmation of the preference for dipolar energy minimization in the nanotube, very few transverse domains were observed for a transverse field, compared with nanowires and parallel-applied fields. The existence of the onion states with non-zero net magnetization, as compared to the vortices in nanowires, also points towards a larger remanence magnetization in nanotubes for perpendicular fields.

In summary, more transverse than vortex domains are modelled at the remanent state of large diameters nanowires and nanotubes for hysteresis loops with a parallel field, and more vortex domains are obtained for the perpendicular ones.

## 5. Conclusions

Polycrystalline Co nanowires and nanotubes with large diameter of 180 nm have been synthesized inside AAO templates. The structure characterization confirms the hexagonal symmetry of crystals with the presence of multiple defects.

The analysis of the experimental hysteresis loops for parallel and perpendicular configuration of the applied field indicates that in both nanowire and nanotube arrays there is an effective longitudinal magnetic anisotropy that is stronger in the case of the nanotube array. In addition, the extremely small remanence for all loops suggests the important role played by magnetostatic interactions.

Micromagnetic simulations were first performed by taking into account the magnetostatic interactions in the mean-field way—as an effective uniaxial easy-plane anisotropy term in a single nanowire. The shape of the modelled hysteresis loops corroborates the strong dipolar interaction between neighboring nanowires. The angular dependence of remanence matches qualitatively well, although the experimental value is lower than the modelled one.

The experimental results are difficult to interpret due to the presence of many factors. In particular, magnetostatic interactions are stronger in the array of nanowires than nanotubes and the difference between the objects can be simply masked by this fact. Simulations were also performed on individual nanowires and nanotubes in order to disentangle different effects, a possibility not experimentally accessible. Complementarity of the experimental findings by micromagnetic simulations permits to understand the differences without many competing effects and the role paid by the sample shapes, without consideration of the magnetostatic interactions. Sequences of vortex and transverse domains forming a helicoidal structure are found at the remanent state. The transverse configurations of the vortex domains for nanotubes consist of onion states, while for the nanowires, the vortices are found to be distorted by the uniaxial anisotropy. A higher proportion of transverse domains is found for parallel applied fields in both structures, while vortices are mainly found for perpendicular applied fields. A larger remanence of the individual nanotube, as compared to an individual nanowire in a parallel configuration, is explained by a larger parallel component of the nanotubes due to an additional influence of the internal surface. This creates an effect of a “larger shape anisotropy” for nanotubes, as compared to nanowires. Thus, two effects contribute to the increased remanence in the array of nanotubes in comparison to nanowires: a smaller interaction strength, due to smaller magnetic volume, and the influence of the nanotube geometry via the existence of the inner surface.

Overall a good agreement is found and a refinement in the model should contribute to increase the accuracy in simulations of the arrays of nanowires and nanotubes.
